# Book Review: The Palgrave Handbook of the Sociology of Work in Europe

**DOI:** 10.3389/fsoc.2019.00062

**Published:** 2019-08-09

**Authors:** Arne L. Kalleberg

**Affiliations:** Department of Sociology, University of North Carolina at Chapel Hill, Chapel Hill, NC, United States

**Keywords:** sociology of work in Europe, organizations, economy, sociology, politics

The Sociology of Work (SoW) has always been an eclectic, often amorphous subfield that draws on the insights, theories, and methods from other areas of Sociology (such as the Sociology of Organizations, Occupations and Professions, Economic Sociology, Social Stratification, Social Psychology, the Sociology of Race and Gender, among others) as well as from other disciplines (such as Economics, Management Science, Industrial Relations, History, Political Science). This reflects the centrality of work to all societies and persons and underscores the utility of studying work for understanding a wide range of concerns, from income inequality, to family dynamics, to the sources of stress and satisfaction. Not surprisingly, then, the topics addressed by the SoW at a particular time and place are intimately related to wider social, economic, and political issues and, as these dynamics change, so too do the subjects that are considered important to study.

The linkages between work and broader societal issues make cross-national studies essential, as such comparative research is needed to illuminate how institutions and cultures shape the nature and consequences of work. The utility of a cross-national approach to studying work was illustrated by Cornfield and Hodson's (2002) exemplary collection of studies from a wide range of countries, from the Americas (Brazil, Canada, Mexico, the United States) to Asia, Africa and the Pacific (Australia, India, Korea, South Africa), and to Europe (France, Belgium, Germany, Great Britain, Portugal, Sweden). In his concluding chapter of that volume, Juan José Castillo underlined the importance of the social context for the topics studied by the SoW in various countries and the methods and theories used. While there was a tendency toward nominal academic convergence, he argued that this was hampered by a “tower of Babel” produced by persistent differences within countries and national scientific communities.

*The Palgrave Handbook of the Sociology of Work in Europe* is one of the few attempts since Cornfield and Hodson (2002) to undertake a comparative analysis of the characteristics and evolution of the SoW in a wide range of countries. It focuses on 11 European countries (the United Kingdom, France, Germany, Italy, Spain, Sweden, Finland, Poland, Hungary, Bulgaria, and Romania). Despite the restriction to Europe, there is more than enough variation among these countries to yield useful contrasts, such as how institutional and cultural factors produce differences among social democratic, liberal market, coordinated market and formerly socialist countries. The editors provide an effective introduction and conclusion to the volume, in which they summarize the main themes of the chapters and the essential characteristics of each of the countries.

The chapters of the *Handbook* highlight the various periods in the development of SoW in each country as well as provide an overview of the main topics studied, the major intellectual traditions, and the exemplary studies and scholars. Each chapter documents how macro socio-economic and political changes in the country influenced the topics studied, thereby demonstrating “the ways in which sociologies of work are socially congruent within the ambit of major socio-economic, historical, and determinate political events” (p. xv).

While the exact periodization of the evolution of the SoW differs somewhat among the 11 European countries, the chapters generally divide its progression since World War II into three broad periods: (1) 1945-1975 (the “Golden Age” of welfare capitalism, Fordism and the rise of social democracy in some countries); (2) 1975-1990s (the decline of the post-war social democratic consensus and Fordism, the rise of globalization and the eclipse of socialism); and (3) 1990s-present (the ascendance of neoliberalism, globalization and financialization, accompanied by the spread of precarious work). There are some exceptions to this, as the SoW in Finland is summarized in terms of two periods, while the chapter on Italy also examines the period before World War II. This general periodization provides a useful framework for showing how studies of work are shaped by the major concerns of especially the dominant social groups at particular times.

We can glean many useful insights from these chapters. For example, they confirm how the topics emphasized in particular periods reflect the power relations and the needs of the most influential actors in society, whether these be productivity, the welfare state, gender and race inequality, immigration, the crisis of capitalism, or precarious work conditions. Moreover, the chapters on Spain, Poland, Hungary, and Bulgaria show how political constraints (produced by right wing or socialist regimes) have inhibited the development of SoW (and in Romania SoW has never emerged as a sub-field within Sociology). The detailed discussions of how the political, economic and social factors affect the development and foci of the sociology of work in these various European countries provide the essential raw materials for the development to a truly cross-national sociology of work, in which macro and meso factors are linked to the study of work and workers.

While the focus on countries as the units of analysis provides the essential raw materials for understanding national divergences and for identifying possible areas of convergence, this emphasis also tends to mask how the dynamics in one country affect those in another. Some of the chapters (e.g., Poland) point to the influence of U.S. sociology on the development of SoW in that country, but a transnational or global approach to explaining the diffusion of ideas and scholarship among countries is not prominent in these chapters (nor is it in sociological research generally). As the spread of globalization and similar patterns of technological transformation are producing greater interdependencies among nations, so too are the issues and approaches adopted by the SoW likely to become more consistent and integrated, despite the “Babelesque” quality of academic communities that are fragmented by different languages and distinctive patterns of conceptual evolution and institutional reframing.

This *Handbook*, with its useful summaries and analyses of SoW in these European countries, is a welcome contribution to the on-going efforts to promote cross-national dialogue about the nature and consequences of work in contemporary societies. It also advances the important goal of establishing economic and political institutions that are able to provide employers and governments with greater flexibility at the same time that they provide workers and their families with job and economic security.

## Author Contributions

The author confirms being the sole contributor of this work and has approved it for publication.

### Conflict of Interest Statement

The author declares that the research was conducted in the absence of any commercial or financial relationships that could be construed as a potential conflict of interest.
